# ProSeq4: A user‐friendly multiplatform program for preparation and analysis of large‐scale DNA polymorphism datasets

**DOI:** 10.1111/1755-0998.13962

**Published:** 2024-04-22

**Authors:** Dmitry A. Filatov

**Affiliations:** ^1^ Department of Biology University of Oxford Oxford UK

**Keywords:** coalescent simulations, data visualisation, DNA polymorphism, file conversion, population genetics, sequence alignment editing, software

## Abstract

Preparation of DNA polymorphism datasets for analysis is an important step in evolutionary genetic and molecular ecology studies. Ever‐growing dataset sizes make this step time consuming, but few convenient software tools are available to facilitate processing of large‐scale datasets including thousands of sequence alignments. Here I report “processor of sequences v4” (proSeq4)—a user‐friendly multiplatform software for preparation and evolutionary genetic analyses of genome‐ or transcriptome‐scale sequence polymorphism datasets. The program has an easy‐to‐use graphic user interface and is designed to process and analyse many thousands of datasets. It supports over two dozen file formats, includes a flexible sequence editor and various tools for data visualization, quality control and most commonly used evolutionary genetic analyses, such as NJ‐phylogeny reconstruction, DNA polymorphism analyses and coalescent simulations. Command line tools (e.g. vcf2fasta) are also provided for easier integration into bioinformatic pipelines. Apart of molecular ecology and evolution research, proSeq4 may be useful for teaching, e.g. for visual illustration of different shapes of phylogenies generated with coalescent simulations in different scenarios. ProSeq4 source code and binaries for Windows, MacOS and Ubuntu are available from https://sourceforge.net/projects/proseq/.

## 1 INTRODUCTION

The availability of genome and/or transcriptome sequence data from multiple individuals of a species provides a lot of power to researches in molecular ecology and evolutionary genetics fields. Many bioinformatic tools have already been developed for various evolutionary genetic analyses of DNA polymorphism data, such as scans for selection (DeGiorgio et al., 2016; Foll & Gaggiotti, 2008), inference of demographic history (Liu & Fu, 2020; Schiffels & Wang, 2020), the extent of genetic exchange between populations or sub‐species and reconstruction of most likely speciation scenarios for closely related species (Excoffier et al., 2021; Gronau et al., 2011; Gutenkunst et al., 2009). However, far fewer convenient open source tools are available for preparation of datasets for evolutionary genetic analyses, particularly so at the genomic or whole‐transcriptome scale.

Modern evolutionary genetic studies are often based on the analysis of DNA polymorphism and/or divergence at thousands of loci (e.g. (Wong & Filatov, 2023)). Datasets of this size can be challenging to prepare, check and correct. A typical workflow in preparation of such datasets includes sequence read mapping to a reference sequence (e.g. with bwa (Li & Durbin, 2010)), processing of resulting read alignments and single nucleotide polymorphism (SNP) calling (e.g. with SAMtools (Li et al., 2009)) and analysis of DNA polymorphism in the resulting VCF (Danecek et al., 2011) file(s). All these steps are done with command‐line tools that are efficient in processing large amounts of data, but offer very limited options to visualize the datasets (e.g. SAMtools view command). Visualization of the datasets to check for any problems and the correction of the identified errors is an important step in workflows of experimental evolutionary genetic studies that is often omitted due to lack of convenient software tools with graphic user interface (GUI) suitable for processing and analysis of large‐scale DNA polymorphism datasets. ProSeq4 was developed to fill this gap in the workflow.

A typical use of proSeq4 in high‐throughput based DNA polymorphism studies is downstream to SNP‐calling: it allows the user to load multisample VCF files (Danecek et al., 2011), convert them to sequence alignments (e.g. in FASTA format), visualize, check, correct, analyse the resulting sequence datasets and convert them to many file formats for downstream analyses in widely used evolutionary genetic programs. Due to its versatility, proSeq4 can also be used for many other purposes, e.g. to load, filter and call consensus for sequence reads from SAM files, as was recently done to reconstruct the sequences for Y‐linked genes (Filatov, 2024). ProSeq4 may also be useful for data processing in low‐throughput sequencing studies, as it includes an editor for sequence chromatograms produced by 2nd generation capillary sequencing machines and it facilitates the assembly of these sequence reads into contigs.

## 2 MATERIALS AND METHODS

ProSeq4 is a 64‐bit program written in Object Pascal. The older 32‐bit versions (Filatov, 2002, 2009) were developed in Borland Delphi using Borland's VCF library that was Windows‐based. ProSeq4 was rewritten in “Lazarus” – a free open source multiplatform Delphi‐like programming environment (https://www.lazarus‐ide.org), using LCL library that is available for many different platforms. ProSeq4 source code, manual, test data and binaries for Windows, MacOS and Ubuntu (v18.04+) are available from https://sourceforge.net/projects/proseq/.

## 3 RESULTS AND DISCUSSION

### 3.1 Data input/output

**FIGURE 1:**
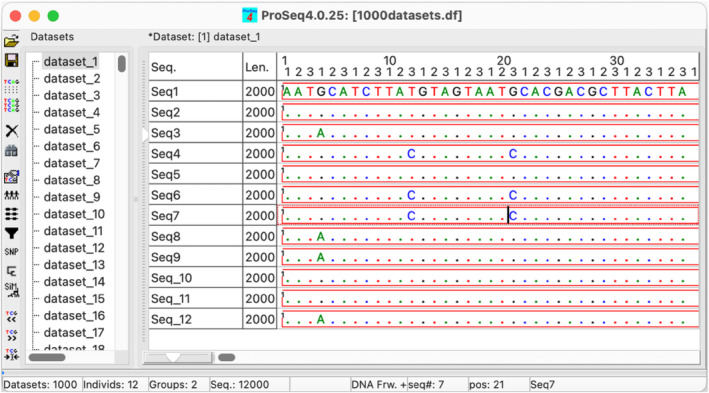
The main window of proSeq4 includes sequence editor, showing the currently active dataset, and the list of datasets in the project at the left. The dots in the sequence alignment denote the same nucleotide as in the first sequence. The sequences can be shown in this ‘dotted mode’ to highlight variable sites, or the ‘standard’ mode with all nucleotides shown. The numbers above the sequences show the position in the alignment and the first, second and third codon positions in the coding region. The red rectangles around the sequences show coding regions assigned to these sequences.

ProSeq4 supports data input and/or output for over two dozen different file formats, including widely used FASTA (Pearson & Lipman, 1988), FASTQ (Cock et al., 2010), SAM (Li et al., 2009) and VCF (Danecek et al., 2011). The data from multiple files, which can be in different file formats, are imported into a multilocus proSeq4 project that can be viewed and edited in the main window of the program (Figure 1).

ProSeq4 was tested with up to 40 thousand datasets (roughly corresponding to the number of genes in an eukaryotic genome) including up to a thousand individuals. Whole genome datasets from multiple individuals can only be handled for relatively small genomes (<150 Mb). For larger genomes the analysis can be done separately for different chromosomal scaffolds. It is also possible to use command line tools included in proSeq4 distribution package to work with large genome‐scale datasets. For example, vcf2fasta program converts VCF files into fasta format (one fasta file per contig or scaffold), while vcfSWpol command line program conducts sliding window DNA polymorphism analysis directly on a multisample VCF file. The command line tools in ProSeq4 package are designed for convenient integration into bioinformatic pipelines. For example, DNA polymorphism analysis in a gzipped VCF file could be done as follows: zcat vcfFile.vcf.gz | ./vcfSWpol ‐i [other options].

A “dataset” in proSeq4 is an alignment of sequences from multiple individuals for a locus or a genomic contig. The project handling multiple datasets is implemented as an internal relational database that allows the user to (optionally) assign sequences to individuals and individuals to populations. Entering this information manually can be tedious for large projects and automatic linking of sequences to individuals, based on similarity of sequence and individual names, is implemented in proSeq4. Functional annotation can (optionally) be assigned to sequences in several ways, including import of annotation from GFF (Pfeifer et al., 2014), BED (Niu et al., 2022) or comma‐delimited CSV files. The resulting multi‐dataset project with functional annotation and population structure can be saved into a “data file” (*.df) that is the binary file format native for proSeq4. Alternatively, the project can be saved as a set of text files, including the list of all files in the project, the population structure file (*.s2i) and fasta files (*.fa) along with their annotation (*.gff). The advantage of the binary *.df file if that it is substantially faster for data input and output operations compared to commonly used text‐based file formats. The data from a proSeq4 project can also be exported into other program‐specific file formats used in various popular evolutionary genetic programs: MEGA (Kumar et al., 2018), PAML (Yang, 2007), DnaSP (Rozas et al., 2017), Structure (Pritchard et al., 2000), Arlequin (Excoffier et al., 2007), fastPhase (Scheet & Stephens, 2006), BayeScan (Foll & Gaggiotti, 2008), SweepFinder (DeGiorgio et al., 2016), GPhoCS (Gronau et al., 2011), bpp (Yang, 2015), MSMC2 (Schiffels & Wang, 2020) and dadi (Gutenkunst et al., 2009). As such, proSeq4 can be used as a convenient converter of file formats for downstream analyses in commonly used evolutionary genetic programs. This proSeq4 functionality is comparable to that of a specialized file conversion tool PGDSpider (Lischer & Excoffier, 2012).

**FIGURE 2:**
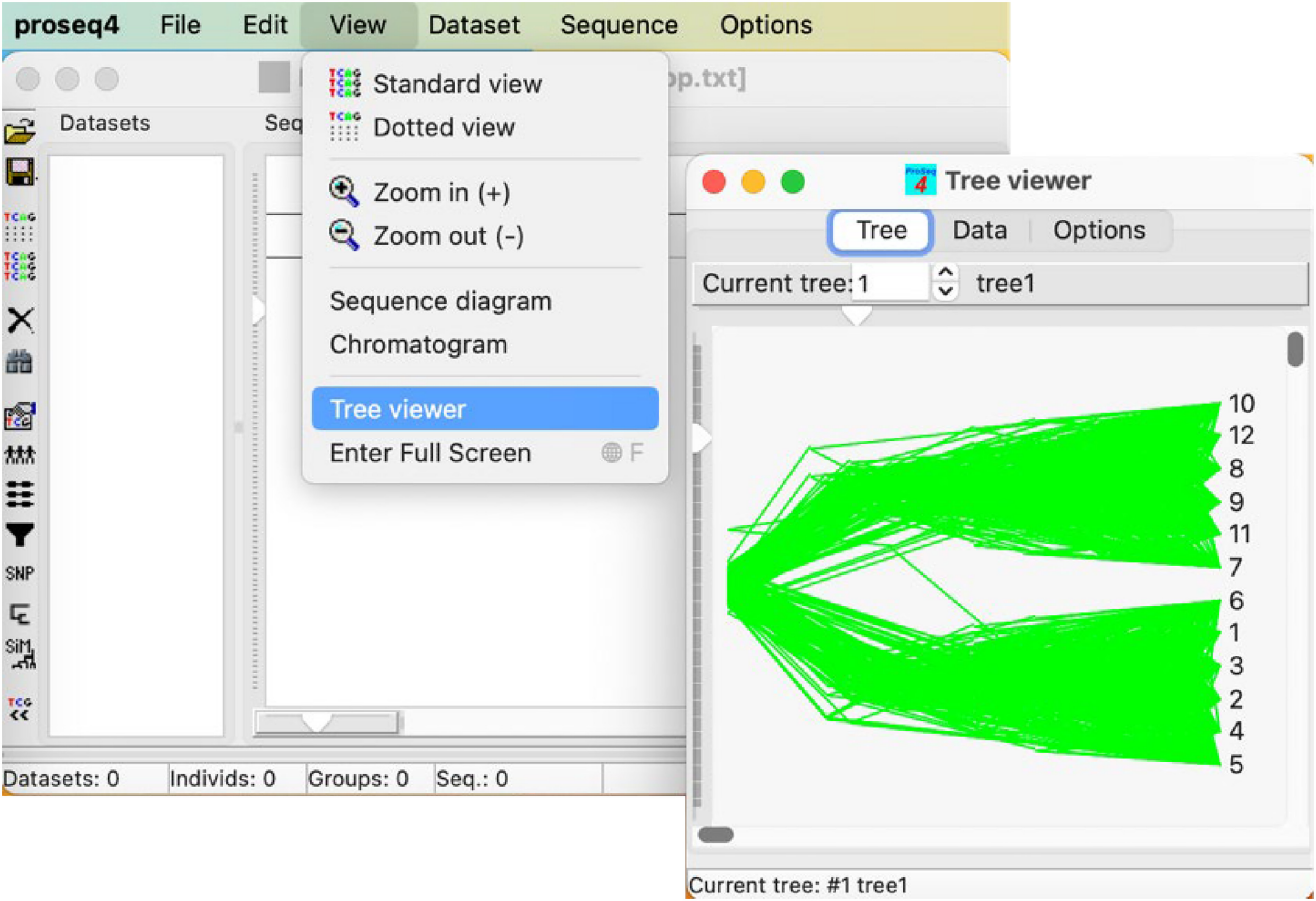
Tree viewer in proSeq4 can visualize multiple phylogenies together. The figure shows the phylogenies from file simTrees_2pop.txt included in the sample data in proSeq4 distribution. These phylogenies were simulated with coalescent simulations tool in proSeq4. The "current tree" in the multiTree mode determines the order of nodes shown at the right.

In addition to various sequence file formats, proSeq4 can open text files with multiple phylogenies in the widely used ‘brackets’ format (e.g. for species A, B and C the phylogeny can be written as ((A,B)C);). The loaded phylogenies are visualized in tree viewer window that can show trees one‐by‐one, or all together in a densiTree (Bouckaert, 2010) ‐like style (Figure 2). The phylogenies can be edited as text in the brackets format on the “Data” page in the tree viewer window, which changes the way the phylogenies are shown on the “Tree” page. Visualization of phylogenies in proSeq4 is more basic than in recently published specialized TreeViewer program (Bianchini & Sanchez‐Baracaldo, 2024), but it is comparable to that in MEGA (Kumar et al., 2018), though the latter cannot show multiple trees at the same time. The key advantage of proSeq4 is in its versatility, facilitating dataset preparation, visualisation and analysis.

### 3.2 Preparation of sequence data for analysis

**FIGURE 3:**
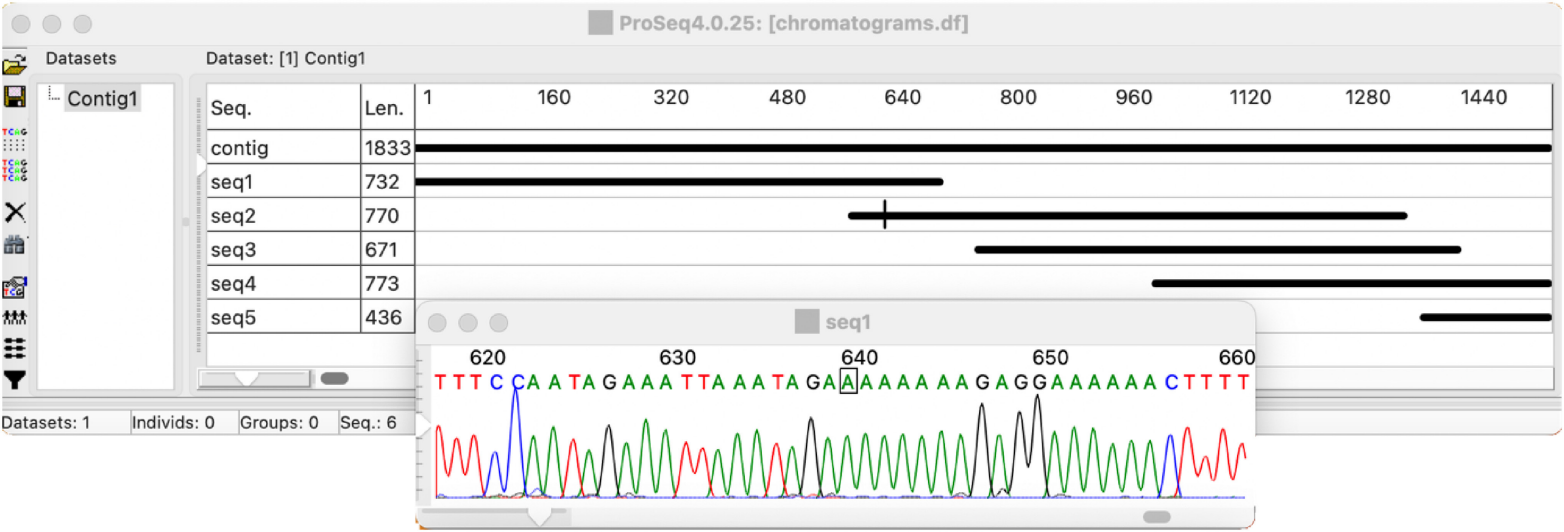
Sequence chromatogram correction and contig assembly.

The main proSeq4 window includes a panel listing all datasets in the project and a sequence editor showing the currently active dataset (Figure 1). It also (optionally) includes an outline sequence diagram at the bottom that shows the entire length of the current sequence, the functional regions assigned to that sequence and the region of the sequence visible in the editor. By default, the sequence diagram is switched off (as on Figure 1), but it can be invoked by “View/Sequence diagram” menu. Zooming out in the sequence editor would also show diagrams for all sequences in the dataset (Figure 3), though the functional annotation would not be shown, unless the row heights in the sequence editor are sufficiently increased (with the vertical slider at the left) to fit the annotations.

The sequence editor allows the user to visualize and manipulate sequences in various ways, including manual sequence editing and aligning, assigning functional regions, sequence searching, reverse‐complementing, changing case, reordering sequences in multiple datasets, sequence trimming, masking and so forth. The editor includes tools for sequence translation, searching for open reading frames and site filtering.

ProSeq4 includes a legacy tool for visualization and correction of sequence chromatograms generated by capillary DNA sequencers. Sequence chromatograms can be loaded into proSeq4 in two file formats: *.ab1 and *.scf. The chromatogram can be visualized in a separate window (Figure 3), which allows the user to correct the sequence inferred from the chromatogram. The sequences with corrected chromatograms can be assembled into a contig and consensus for the contig can be called in the main proSeq4 editor window (Figure 3). This proSeq4 functionality provides a free alternative to such commercial software as Sequencher (GeneCodes Corp.).

ProSeq4 includes several tools for quality control of the data in the project. In particular, it can create summary reports for the datasets, sequences, coding regions and missing data in the project. This allows the user to identify errors with assignment of coding regions, such as the presence of premature stop codons and the regions with possible misalignment. For example, the “Gaps and missing data report” helps to identify and remove individuals with too much missing data in the assigned sequences. The site filtering tool allows the user to automatically filter alignment positions in all datasets in the project. For example, it is possible to exclude first and second codon positions as well as the sites with indels or missing data.

### 3.3 Phylogenetic analyses

Phylogeny reconstructions in proSeq4 are limited to Neighbour‐Joining (Saitou & Nei, 1987) with the phylogenetic distance represented as the number of nucleotide differences, Jukes‐Cantor (Jukes & Cantor, 1969) or Kimura's 2 parameter (Kimura, 1980) distance. In one go the phylogenies can be reconstructed for all datasets in the project and the resulting phylogenies are visualized in the tree viewer (Figure 2). This allows the user to visually assess the extent of incongruence between the phylogenies reconstructed for different datasets (e.g. for different genes in the genome) and to identify potentially problematic datasets, e.g. containing highly divergent sequences that could indicate paralogy or problems with alignment. Although proSeq4 functionality for phylogenetic reconstructions is quite basic, it helps with checking and visually exploring the data before more detailed downstream analyses in specialized phylogenetic programs such as MEGA (Kumar et al., 2018), PhyML (Guindon & Gascuel, 2003) and PAML (Yang, 2007).

### 3.4 Population genetic analyses

ProSeq4 implements many of the widely used DNA polymorphism summary statistics, such as average nucleotide diversity π (Nei, 1987), Watterson's θ (Watterson, 1975), Tajima's D (Tajima, 1989), Kelly's *Z*_nS_ (Kelly, 1997) etc, which can be calculated for the current, or for all datasets in the project. These statistics can be calculated in a sliding window of given size to analyse the distribution of level and patterns of DNA polymorphism along the sequence length. If coding region(s) were assigned to a dataset, the statistics are calculated separately for silent and non‐silent sites. An alternative way to analyse polymorphism at different types of sites is to run the analysis on pre‐filtered datasets created with “Dataset/Filter datasets/Filter sites in all datasets” menu. If the project includes population structure (assigned with “Edit/Edit groups and individs” menu) with at least two populations (or groups of individuals), the program also calculates population subdivision statistics, such as the numbers of shared and fixed sites, *F*_st_ and *K*_st_ (Hudson et al., 1992), and their significance is tested with permutation (that is, randomly swapping sequences between populations). The population genetic analyses implemented in proSeq4 are roughly similar to that in recent versions of DNAsp after an upgrade enabling that program to analyse multiple datasets (Rozas et al., 2017). However, unlike DNAsp, proSeq4 is designed not only to analyse but also to facilitate preparation of multigenic datasets for analyses.

**FIGURE 4:**
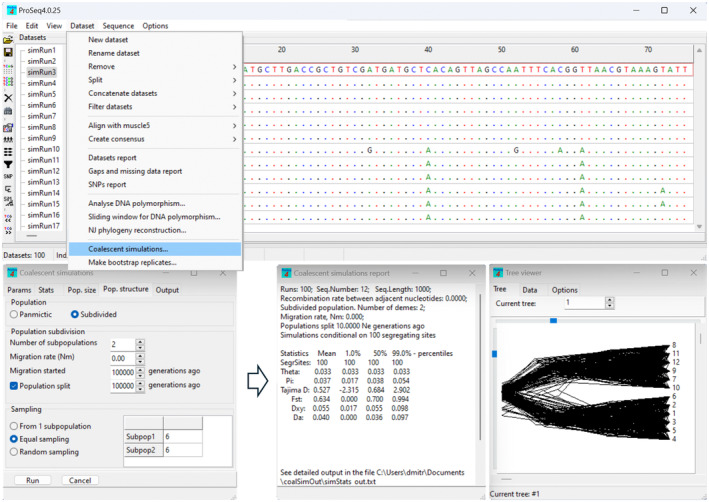
Coalescent simulations tool in proSeq4 can be used to generate critical values for the statistics and simulate gene trees and datasets under various demographic scenarios.

A convenient way to obtain bespoke critical values for statistics in the particular dataset is to run coalescent simulations with the same size and level of polymorphism as in the experimental dataset (Hudson, 1992). For this purpose, proSeq4 includes coalescent simulations tool that can run simulations under various demographic scenarios, including population size change and population subdivision. The output of coalescent simulations includes the critical values for the statistics of interest as well as (optionally) the simulated datasets in the form of sequence alignments (Figure 4). These simulated datasets can be saved in any of the supported file formats for downstream analyses in other programs. Furthermore, it is possible to save and/or visualize the genealogies (Figure 4) generated as part of the coalescent simulations process (Hudson, 1992). This flexibility and versatility of proSeq4 is unmatched by other programs for coalescent simulations (e.g. Hudson's ms program (Hudson, 1992) or coalescent simulations tool in DNAsp (Rozas et al., 2017)).

### 3.5 The use of proSeq4 for teaching

Students often find evolutionary genetic concepts counterintuitive. Dataset visualization facilitates understanding and illustrates the analyses that are done during evolutionary genetic practicals. Coalescent theory, with its upside down phylogenies and time running backwards (Hudson, 1992), is one of the most difficult topics for students to understand. Visualization of simulated phylogenies and datasets can help students to better grasp the concepts and the use of coalescent theory in evolutionary genetics. To this end, proSeq4 includes a tool (Figure 4) to run coalescent simulations and to simulate datasets under various scenarios. The simulated phylogenies can be visualized in tree viewer tool in proSeq4 (Figure 2). The simulated DNA polymorphism datasets (Figure 4) can be viewed in proSeq4 sequence editor, and these datasets can be used to calculate a statistic of user's choice (e.g. Tajima's D (Tajima, 1989)), which generates the null distribution of that statistic expected under the simulation scenario. This serves as a visual illustration for students how coalescent simulations can be used to generate a bespoke null distribution for the specific dataset, which is a common use of coalescent theory in experimental evolutionary genetics.

## 4 CONCLUSIONS

ProSeq4 is a versatile program for dataset preparation and most common evolutionary genetic analyses. It can be useful at various steps in the workflows of molecular ecology and evolution studies involving compilation, quality control, visualization and analysis of DNA polymorphism datasets. The program facilitates handling and efficient analysis of large‐scale high‐throughput datasets but also includes tools for editing of data generated by legacy 2nd generation sequencing machines. Large number of file formats supported by proSeq4 makes it useful as a convenient and powerful file conversion tool. Given the versatility of this user friendly GUI program, it is likely to be useful for many users in Ecology and Evolution fields.

## conflict of interest statement

The author declared no conflict of interest.

## Data Availability

ProSeq4 source code and binaries for Windows, Mac and Ubuntu are available from https://sourceforge.net/projects/proseq/.
